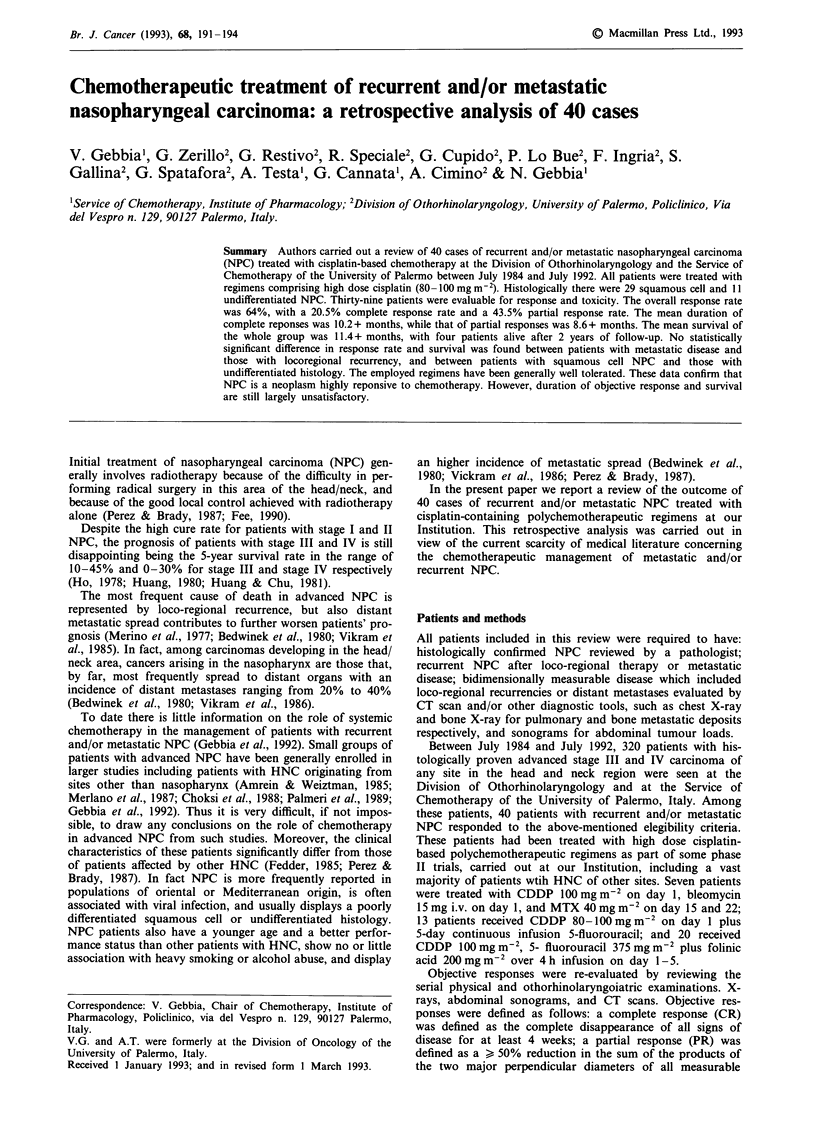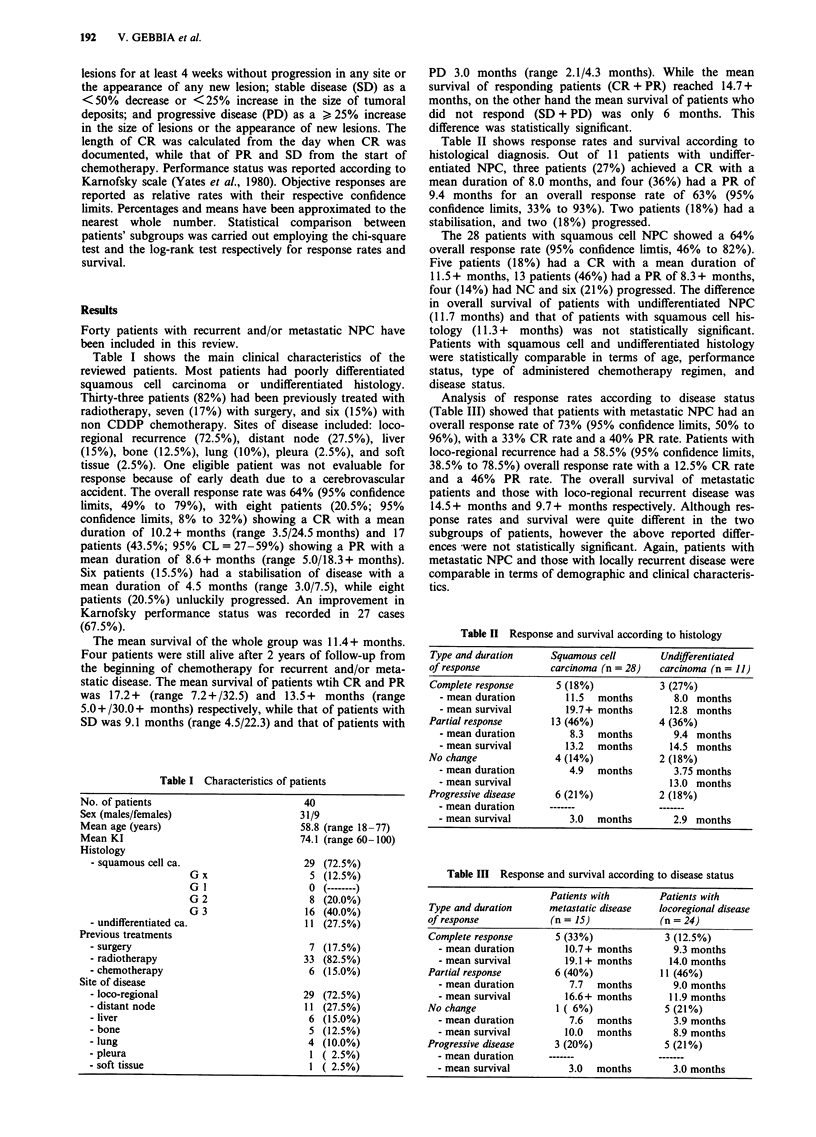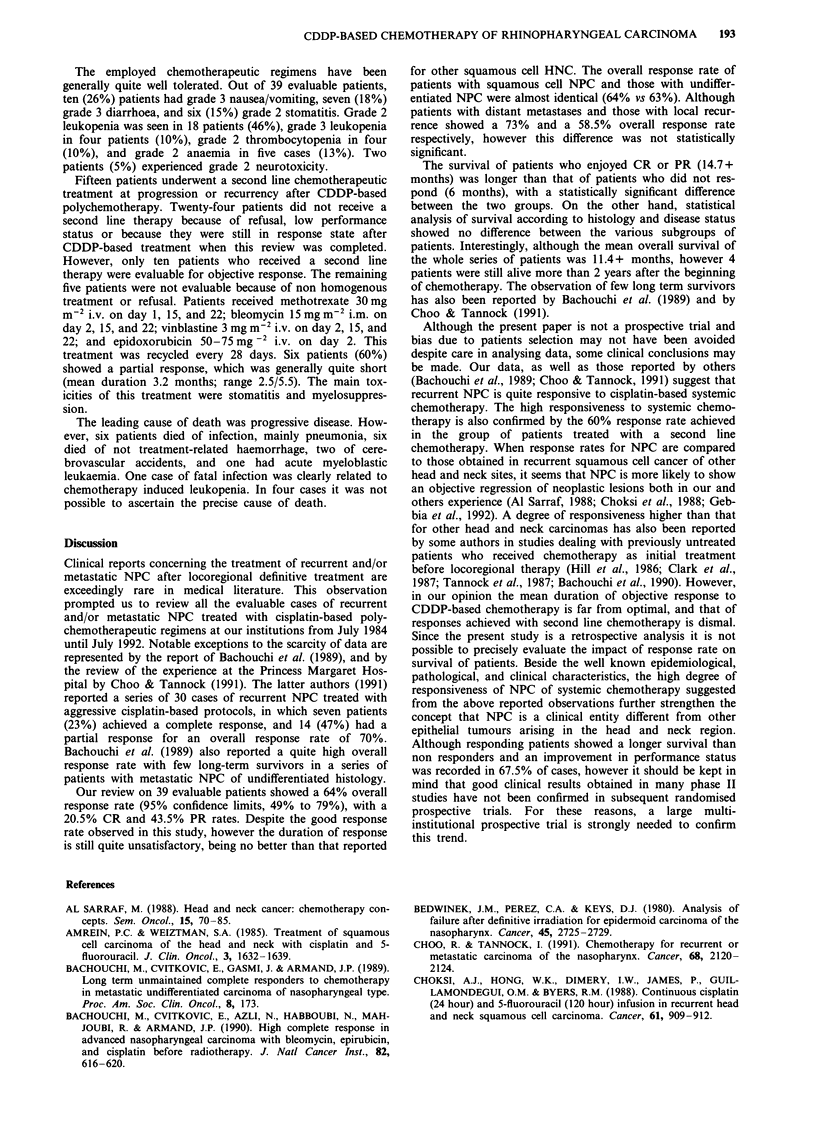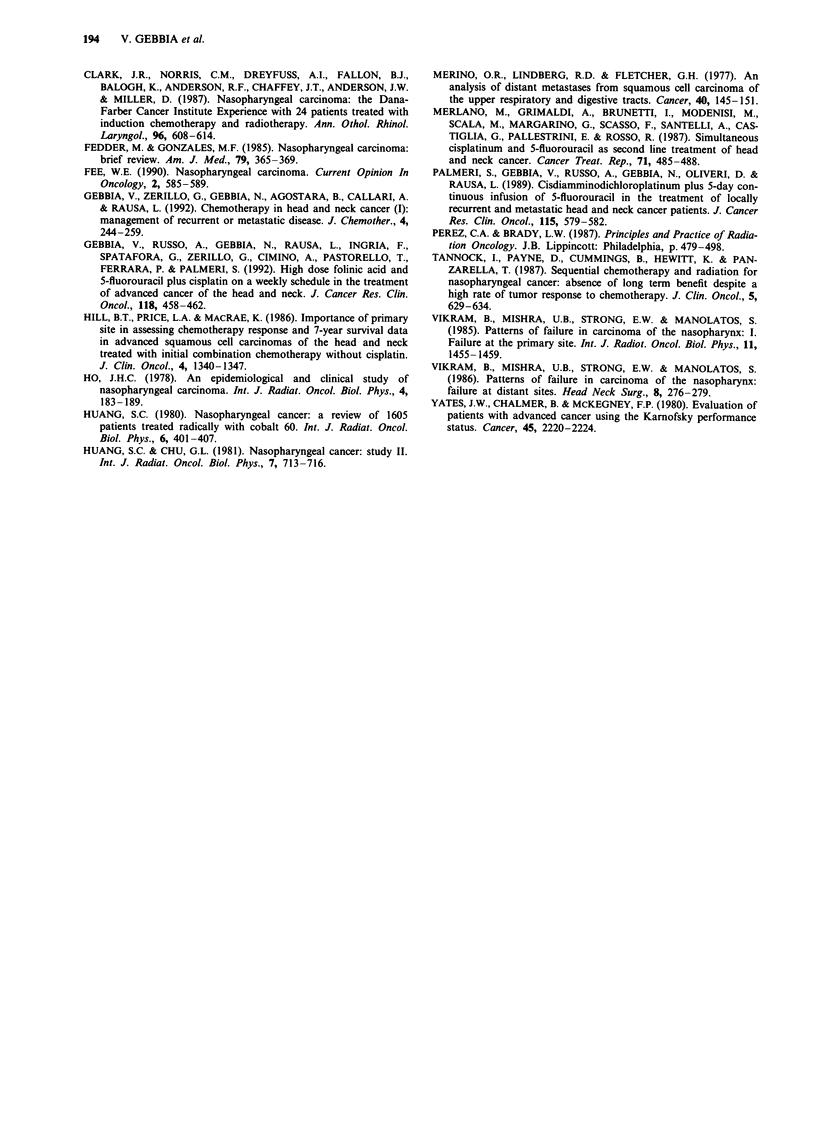# Chemotherapeutic treatment of recurrent and/or metastatic nasopharyngeal carcinoma: a retrospective analysis of 40 cases.

**DOI:** 10.1038/bjc.1993.312

**Published:** 1993-07

**Authors:** V. Gebbia, G. Zerillo, G. Restivo, R. Speciale, G. Cupido, P. Lo Bue, F. Ingria, S. Gallina, G. Spatafora, A. Testa

**Affiliations:** Service of Chemotherapy, Institute of Pharmacology, Palermo, Italy.

## Abstract

Authors carried out a review of 40 cases of recurrent and/or metastatic nasopharyngeal carcinoma (NPC) treated with cisplatin-based chemotherapy at the Division of Othorhinolaryngology and the Service of Chemotherapy of the University of Palermo between July 1984 and July 1992. All patients were treated with regimens comprising high dose cisplatin (80-100 mg m-2). Histologically there were 29 squamous cell and 11 undifferentiated NPC. Thirty-nine patients were evaluable for response and toxicity. The overall response rate was 64%, with a 20.5% complete response rate and a 43.5% partial response rate. The mean duration of complete responses was 10.2+months, while that of partial responses was 8.6+months. The mean survival of the whole group was 11.4+months, with four patients alive after 2 years of follow-up. No statistically significant difference in response rate and survival was found between patients with metastatic disease and those with locoregional recurrency, and between patients with squamous cell NPC and those with undifferentiated histology. The employed regimens have been generally well tolerated. These data confirm that NPC is a neoplasm highly responsive to chemotherapy. However, duration of objective response and survival are still largely unsatisfactory.


Br. J. Cancer (1993), 68, 191–194 © Macmillan Press Ltd., 1993

### Chemotherapeutic treatment of recurrent and/or metastatic nasopharyngeal carcinoma: a retrospective analysis of 40 cases

V. Gebbia[1], G. Zerillo[2], G. Restivo[2], R. Speciale[2], G. Cupido[2], P. Lo Bue[2], F. Ingria[2], S. Gallina[2], G. Spatafora[2], A. Testa[1], G. Cannata[1], A. Cimino[2] & N. Gebbia[1]

[1]*Service of Chemotherapy, Institute of Pharmacology;* [2]*Division of Othorhinolaryngology, University of Palermo, Policlinico, Via del Vespro n. 129, 90127 Palermo, Italy.*

**Summary** Authors carried out a review of 40 cases of recurrent and/or metastatic nasopharyngeal carcinoma (NPC) treated with cisplatin-based chemotherapy at the Division of Othorhinolaryngology and the Service of Chemotherapy of the University of Palermo between July 1984 and July 1992. All patients were treated with regimens comprising high dose cisplatin (80–100 mg m$^{-2}$). Histologically there were 29 squamous cell and 11 undifferentiated NPC. Thirty-nine patients were evaluable for response and toxicity. The overall response rate was 64%, with a 20.5% complete response rate and a 43.5% partial response rate. The mean duration of complete reponses was 10.2+ months, while that of partial responses was 8.6+ months. The mean survival of the whole group was 11.4+ months, with four patients alive after 2 years of follow-up. No statistically significant difference in response rate and survival was found between patients with metastatic disease and those with locoregional recurrency, and between patients with squamous cell NPC and those with undifferentiated histology. The employed regimens have been generally well tolerated. These data confirm that NPC is a neoplasm highly reponsive to chemotherapy. However, duration of objective response and survival are still largely unsatisfactory.

Initial treatment of nasopharyngeal carcinoma (NPC) generally involves radiotherapy because of the difficulty in performing radical surgery in this area of the head/neck, and because of the good local control achieved with radiotherapy alone (Perez & Brady, 1987; Fee, 1990).

Despite the high cure rate for patients with stage I and II NPC, the prognosis of patients with stage III and IV is still disappointing being the 5-year survival rate in the range of 10–45% and 0–30% for stage III and stage IV respectively (Ho, 1978; Huang, 1980; Huang & Chu, 1981).

The most frequent cause of death in advanced NPC is represented by loco-regional recurrence, but also distant metastatic spread contributes to further worsen patients' prognosis (Merino *et al.*, 1977; Bedwinek *et al.*, 1980; Vikram *et al.*, 1985). In fact, among carcinomas developing in the head/neck area, cancers arising in the nasopharynx are those that, by far, most frequently spread to distant organs with an incidence of distant metastases ranging from 20% to 40% (Bedwinek *et al.*, 1980; Vikram *et al.*, 1986).

To date there is little information on the role of systemic chemotherapy in the management of patients with recurrent and/or metastatic NPC (Gebbia *et al.*, 1992). Small groups of patients with advanced NPC have been generally enrolled in larger studies including patients with HNC originating from sites other than nasopharynx (Amrein & Weiztman, 1985; Merlano *et al.*, 1987; Choksi *et al.*, 1988; Palmeri *et al.*, 1989; Gebbia *et al.*, 1992). Thus it is very difficult, if not impossible, to draw any conclusions on the role of chemotherapy in advanced NPC from such studies. Moreover, the clinical characteristics of these patients significantly differ from those of patients affected by other HNC (Fedder, 1985; Perez & Brady, 1987). In fact NPC is more frequently reported in populations of oriental or Mediterranean origin, is often associated with viral infection, and usually displays a poorly differentiated squamous cell or undifferentiated histology. NPC patients also have a younger age and a better performance status than other patients with HNC, show no or little association with heavy smoking or alcohol abuse, and display an higher incidence of metastatic spread (Bedwinek *et al.*, 1980; Vickram *et al.*, 1986; Perez & Brady, 1987).

In the present paper we report a review of the outcome of 40 cases of recurrent and/or metastatic NPC treated with cisplatin-containing polychemotherapeutic regimens at our Institution. This retrospective analysis was carried out in view of the current scarcity of medical literature concerning the chemotherapeutic management of metastatic and/or recurrent NPC.

### Patients and methods

All patients included in this review were required to have: histologically confirmed NPC reviewed by a pathologist; recurrent NPC after loco-regional therapy or metastatic disease; bidimensionally measurable disease which included loco-regional recurrencies or distant metastases evaluated by CT scan and/or other diagnostic tools, such as chest X-ray and bone X-ray for pulmonary and bone metastatic deposits respectively, and sonograms for abdominal tumour loads.

Between July 1984 and July 1992, 320 patients with histologically proven advanced stage III and IV carcinoma of any site in the head and neck region were seen at the Division of Othorhinolaryngology and at the Service of Chemotherapy of the University of Palermo, Italy. Among these patients, 40 patients with recurrent and/or metastatic NPC responded to the above-mentioned elegibility criteria. These patients had been treated with high dose cisplatin-based polychemotherapeutic regimens as part of some phase II trials, carried out at our Institution, including a vast majority of patients wtih HNC of other sites. Seven patients were treated with CDDP 100 mg m$^{-2}$ on day 1, bleomycin 15 mg i.v. on day 1, and MTX 40 mg m$^{-2}$ on day 15 and 22; 13 patients received CDDP 80–100 mg m$^{-2}$ on day 1 plus 5-day continuous infusion 5-fluorouracil; and 20 received CDDP 100 mg m$^{-2}$, 5- fluorouracil 375 mg m$^{-2}$ plus folinic acid 200 mg m$^{-2}$ over 4 h infusion on day 1–5.

Objective responses were re-evaluated by reviewing the serial physical and othorhinolaryngoiatric examinations. X-rays, abdominal sonograms, and CT scans. Objective responses were defined as follows: a complete response (CR) was defined as the complete disappearance of all signs of disease for at least 4 weeks; a partial response (PR) was defined as a $\geqslant 50\%$ reduction in the sum of the products of the two major perpendicular diameters of all measurable

Correspondence: V. Gebbia, Chair of Chemotherapy, Institute of Pharmacology, Policlinico, via del Vespro n. 129, 90127 Palermo, Italy.
V.G. and A.T. were formerly at the Division of Oncology of the University of Palermo, Italy.
Received 1 January 1993; and in revised form 1 March 1993.

lesions for at least 4 weeks without progression in any site or the appearance of any new lesion; stable disease (SD) as a <50% decrease or <25% increase in the size of tumoral deposits; and progressive disease (PD) as a ≥25% increase in the size of lesions or the appearance of new lesions. The length of CR was calculated from the day when CR was documented, while that of PR and SD from the start of chemotherapy. Performance status was reported according to Karnofsky scale (Yates *et al.*, 1980). Objective responses are reported as relative rates with their respective confidence limits. Percentages and means have been approximated to the nearest whole number. Statistical comparison between patients' subgroups was carried out employing the chi-square test and the log-rank test respectively for response rates and survival.

### Results

Forty patients with recurrent and/or metastatic NPC have been included in this review.

Table I shows the main clinical characteristics of the reviewed patients. Most patients had poorly differentiated squamous cell carcinoma or undifferentiated histology. Thirty-three patients (82%) had been previously treated with radiotherapy, seven (17%) with surgery, and six (15%) with non CDDP chemotherapy. Sites of disease included: loco-regional recurrence (72.5%), distant node (27.5%), liver (15%), bone (12.5%), lung (10%), pleura (2.5%), and soft tissue (2.5%). One eligible patient was not evaluable for response because of early death due to a cerebrovascular accident. The overall response rate was 64% (95% confidence limits, 49% to 79%), with eight patients (20.5%; 95% confidence limits, 8% to 32%) showing a CR with a mean duration of 10.2+ months (range 3.5/24.5 months) and 17 patients (43.5%; 95% CL = 27–59%) showing a PR with a mean duration of 8.6+ months (range 5.0/18.3+ months). Six patients (15.5%) had a stabilisation of disease with a mean duration of 4.5 months (range 3.0/7.5), while eight patients (20.5%) unluckily progressed. An improvement in Karnofsky performance status was recorded in 27 cases (67.5%).

The mean survival of the whole group was 11.4+ months. Four patients were still alive after 2 years of follow-up from the beginning of chemotherapy for recurrent and/or metastatic disease. The mean survival of patients wtih CR and PR was 17.2+ (range 7.2+/32.5) and 13.5+ months (range 5.0+/30.0+ months) respectively, while that of patients with SD was 9.1 months (range 4.5/22.3) and that of patients with PD 3.0 months (range 2.1/4.3 months). While the mean survival of responding patients (CR + PR) reached 14.7+ months, on the other hand the mean survival of patients who did not respond (SD + PD) was only 6 months. This difference was statistically significant.

Table II shows response rates and survival according to histological diagnosis. Out of 11 patients with undifferentiated NPC, three patients (27%) achieved a CR with a mean duration of 8.0 months, and four (36%) had a PR of 9.4 months for an overall response rate of 63% (95% confidence limits, 33% to 93%). Two patients (18%) had a stabilisation, and two (18%) progressed.

The 28 patients with squamous cell NPC showed a 64% overall response rate (95% confidence limtis, 46% to 82%). Five patients (18%) had a CR with a mean duration of 11.5+ months, 13 patients (46%) had a PR of 8.3+ months, four (14%) had NC and six (21%) progressed. The difference in overall survival of patients with undifferentiated NPC (11.7 months) and that of patients with squamous cell histology (11.3+ months) was not statistically significant. Patients with squamous cell and undifferentiated histology were statistically comparable in terms of age, performance status, type of administered chemotherapy regimen, and disease status.

Analysis of response rates according to disease status (Table III) showed that patients with metastatic NPC had an overall response rate of 73% (95% confidence limits, 50% to 96%), with a 33% CR rate and a 40% PR rate. Patients with loco-regional recurrence had a 58.5% (95% confidence limits, 38.5% to 78.5%) overall response rate with a 12.5% CR rate and a 46% PR rate. The overall survival of metastatic patients and those with loco-regional recurrent disease was 14.5+ months and 9.7+ months respectively. Although response rates and survival were quite different in the two subgroups of patients, however the above reported differences were not statistically significant. Again, patients with metastatic NPC and those with locally recurrent disease were comparable in terms of demographic and clinical characteristics.

**Table I** Characteristics of patients

| | |
|---|---|
| No. of patients | 40 |
| Sex (males/females) | 31/9 |
| Mean age (years) | 58.8 (range 18–77) |
| Mean KI | 74.1 (range 60–100) |
| Histology | |
| - squamous cell ca. | 29 (72.5%) |
| G x | 5 (12.5%) |
| G 1 | 0 (--------) |
| G 2 | 8 (20.0%) |
| G 3 | 16 (40.0%) |
| - undifferentiated ca. | 11 (27.5%) |
| Previous treatments | |
| - surgery | 7 (17.5%) |
| - radiotherapy | 33 (82.5%) |
| - chemotherapy | 6 (15.0%) |
| Site of disease | |
| - loco-regional | 29 (72.5%) |
| - distant node | 11 (27.5%) |
| - liver | 6 (15.0%) |
| - bone | 5 (12.5%) |
| - lung | 4 (10.0%) |
| - pleura | 1 ( 2.5%) |
| - soft tissue | 1 ( 2.5%) |

**Table II** Response and survival according to histology

| *Type and duration of response* | *Squamous cell carcinoma* (n = *28)* | *Undifferentiated carcinoma (*n = *11)* |
|---|---|---|
| *Complete response* | 5 (18%) | 3 (27%) |
| - mean duration | 11.5 months | 8.0 months |
| - mean survival | 19.7+ months | 12.8 months |
| *Partial response* | 13 (46%) | 4 (36%) |
| - mean duration | 8.3 months | 9.4 months |
| - mean survival | 13.2 months | 14.5 months |
| *No change* | 4 (14%) | 2 (18%) |
| - mean duration | 4.9 months | 3.75 months |
| - mean survival | | 13.0 months |
| *Progressive disease* | 6 (21%) | 2 (18%) |
| - mean duration | ------- | ------- |
| - mean survival | 3.0 months | 2.9 months |

**Table III** Response and survival according to disease status

| *Type and duration of response* | *Patients with metastatic disease (*n = *15)* | *Patients with locoregional disease (*n = *24)* |
|---|---|---|
| *Complete response* | 5 (33%) | 3 (12.5%) |
| - mean duration | 10.7+ months | 9.3 months |
| - mean survival | 19.1+ months | 14.0 months |
| *Partial response* | 6 (40%) | 11 (46%) |
| - mean duration | 7.7 months | 9.0 months |
| - mean survival | 16.6+ months | 11.9 months |
| *No change* | 1 ( 6%) | 5 (21%) |
| - mean duration | 7.6 months | 3.9 months |
| - mean survival | 10.0 months | 8.9 months |
| *Progressive disease* | 3 (20%) | 5 (21%) |
| - mean duration | ------- | ------- |
| - mean survival | 3.0 months | 3.0 months |

The employed chemotherapeutic regimens have been generally quite well tolerated. Out of 39 evaluable patients, ten (26%) patients had grade 3 nausea/vomiting, seven (18%) grade 3 diarrhoea, and six (15%) grade 2 stomatitis. Grade 2 leukopenia was seen in 18 patients (46%), grade 3 leukopenia in four patients (10%), grade 2 thrombocytopenia in four (10%), and grade 2 anaemia in five cases (13%). Two patients (5%) experienced grade 2 neurotoxicity.

Fifteen patients underwent a second line chemotherapeutic treatment at progression or recurrency after CDDP-based polychemotherapy. Twenty-four patients did not receive a second line therapy because of refusal, low performance status or because they were still in response state after CDDP-based treatment when this review was completed. However, only ten patients who received a second line therapy were evaluable for objective response. The remaining five patients were not evaluable because of non homogenous treatment or refusal. Patients received methotrexate 30 mg $m^{-2}$ i.v. on day 1, 15, and 22; bleomycin 15 mg $m^{-2}$ i.m. on day 2, 15, and 22; vinblastine 3 mg $m^{-2}$ i.v. on day 2, 15, and 22; and epidoxorubicin 50–75 mg $^{-2}$ i.v. on day 2. This treatment was recycled every 28 days. Six patients (60%) showed a partial response, which was generally quite short (mean duration 3.2 months; range 2.5/5.5). The main toxicities of this treatment were stomatitis and myelosuppression.

The leading cause of death was progressive disease. However, six patients died of infection, mainly pneumonia, six died of not treatment-related haemorrhage, two of cerebrovascular accidents, and one had acute myeloblastic leukaemia. One case of fatal infection was clearly related to chemotherapy induced leukopenia. In four cases it was not possible to ascertain the precise cause of death.

### Discussion

Clinical reports concerning the treatment of recurrent and/or metastatic NPC after locoregional definitive treatment are exceedingly rare in medical literature. This observation prompted us to review all the evaluable cases of recurrent and/or metastatic NPC treated with cisplatin-based polychemotherapeutic regimens at our institutions from July 1984 until July 1992. Notable exceptions to the scarcity of data are represented by the report of Bachouchi *et al.* (1989), and by the review of the experience at the Princess Margaret Hospital by Choo & Tannock (1991). The latter authors (1991) reported a series of 30 cases of recurrent NPC treated with aggressive cisplatin-based protocols, in which seven patients (23%) achieved a complete response, and 14 (47%) had a partial response for an overall response rate of 70%. Bachouchi *et al.* (1989) also reported a quite high overall response rate with few long-term survivors in a series of patients with metastatic NPC of undifferentiated histology.

Our review on 39 evaluable patients showed a 64% overall response rate (95% confidence limits, 49% to 79%), with a 20.5% CR and 43.5% PR rates. Despite the good response rate observed in this study, however the duration of response is still quite unsatisfactory, being no better than that reported for other squamous cell HNC. The overall response rate of patients with squamous cell NPC and those with undifferentiated NPC were almost identical (64% *vs* 63%). Although patients with distant metastases and those with local recurrence showed a 73% and a 58.5% overall response rate respectively, however this difference was not statistically significant.

The survival of patients who enjoyed CR or PR (14.7+ months) was longer than that of patients who did not respond (6 months), with a statistically significant difference between the two groups. On the other hand, statistical analysis of survival according to histology and disease status showed no difference between the various subgroups of patients. Interestingly, although the mean overall survival of the whole series of patients was 11.4+ months, however 4 patients were still alive more than 2 years after the beginning of chemotherapy. The observation of few long term survivors has also been reported by Bachouchi *et al.* (1989) and by Choo & Tannock (1991).

Although the present paper is not a prospective trial and bias due to patients selection may not have been avoided despite care in analysing data, some clinical conclusions may be made. Our data, as well as those reported by others (Bachouchi *et al.*, 1989; Choo & Tannock, 1991) suggest that recurrent NPC is quite responsive to cisplatin-based systemic chemotherapy. The high responsiveness to systemic chemotherapy is also confirmed by the 60% response rate achieved in the group of patients treated with a second line chemotherapy. When response rates for NPC are compared to those obtained in recurrent squamous cell cancer of other head and neck sites, it seems that NPC is more likely to show an objective regression of neoplastic lesions both in our and others experience (Al Sarraf, 1988; Choksi *et al.*, 1988; Gebbia *et al.*, 1992). A degree of responsiveness higher than that for other head and neck carcinomas has also been reported by some authors in studies dealing with previously untreated patients who received chemotherapy as initial treatment before locoregional therapy (Hill *et al.*, 1986; Clark *et al.*, 1987; Tannock *et al.*, 1987; Bachouchi *et al.*, 1990). However, in our opinion the mean duration of objective response to CDDP-based chemotherapy is far from optimal, and that of responses achieved with second line chemotherapy is dismal. Since the present study is a retrospective analysis it is not possible to precisely evaluate the impact of response rate on survival of patients. Beside the well known epidemiological, pathological, and clinical characteristics, the high degree of responsiveness of NPC of systemic chemotherapy suggested from the above reported observations further strengthen the concept that NPC is a clinical entity different from other epithelial tumours arising in the head and neck region. Although responding patients showed a longer survival than non responders and an improvement in performance status was recorded in 67.5% of cases, however it should be kept in mind that good clinical results obtained in many phase II studies have not been confirmed in subsequent randomised prospective trials. For these reasons, a large multi-institutional prospective trial is strongly needed to confirm this trend.

### References

AL SARRAF, M. (1988). Head and neck cancer: chemotherapy concepts. *Sem. Oncol.*, **15**, 70–85.

AMREIN, P.C. & WEIZTMAN, S.A. (1985). Treatment of squamous cell carcinoma of the head and neck with cisplatin and 5-fluorouracil. *J. Clin. Oncol.*, **3**, 1632–1639.

BACHOUCHI, M., CVITKOVIC, E., GASMI, J. & ARMAND, J.P. (1989). Long term unmaintained complete responders to chemotherapy in metastatic undifferentiated carcinoma of nasopharyngeal type. *Proc. Am. Soc. Clin. Oncol.*, **8**, 173.

BACHOUCHI, M., CVITKOVIC, E., AZLI, N., HABBOUBI, N., MAHJOUBI, R. & ARMAND, J.P. (1990). High complete response in advanced nasopharyngeal carcinoma with bleomycin, epirubicin, and cisplatin before radiotherapy. *J. Natl Cancer Inst.*, **82**, 616–620.

BEDWINEK, J.M., PEREZ, C.A. & KEYS, D.J. (1980). Analysis of failure after definitive irradiation for epidermoid carcinoma of the nasopharynx. *Cancer*, **45**, 2725–2729.

CHOO, R. & TANNOCK, I. (1991). Chemotherapy for recurrent or metastatic carcinoma of the nasopharynx. *Cancer*, **68**, 2120–2124.

CHOKSI, A.J., HONG, W.K., DIMERY, I.W., JAMES, P., GUILLAMONDEGUI, O.M. & BYERS, R.M. (1988). Continuous cisplatin (24 hour) and 5-fluorouracil (120 hour) infusion in recurrent head and neck squamous cell carcinoma. *Cancer*, **61**, 909–912.

CLARK, J.R., NORRIS, C.M., DREYFUSS, A.I., FALLON, B.J., BALOGH, K., ANDERSON, R.F., CHAFFEY, J.T., ANDERSON, J.W. & MILLER, D. (1987). Nasopharyngeal carcinoma: the Dana-Farber Cancer Institute Experience with 24 patients treated with induction chemotherapy and radiotherapy. *Ann. Othol. Rhinol. Laryngol.*, **96**, 608–614.

FEDDER, M. & GONZALES, M.F. (1985). Nasopharyngeal carcinoma: brief review. *Am. J. Med.*, **79**, 365–369.

FEE, W.E. (1990). Nasopharyngeal carcinoma. *Current Opinion In Oncology*, **2**, 585–589.

GEBBIA, V., ZERILLO, G., GEBBIA, N., AGOSTARA, B., CALLARI, A. & RAUSA, L. (1992). Chemotherapy in head and neck cancer (I): management of recurrent or metastatic disease. *J. Chemother.*, **4**, 244–259.

GEBBIA, V., RUSSO, A., GEBBIA, N., RAUSA, L., INGRIA, F., SPATAFORA, G., ZERILLO, G., CIMINO, A., PASTORELLO, T., FERRARA, P. & PALMERI, S. (1992). High dose folinic acid and 5-fluorouracil plus cisplatin on a weekly schedule in the treatment of advanced cancer of the head and neck. *J. Cancer Res. Clin. Oncol.*, **118**, 458–462.

HILL, B.T., PRICE, L.A. & MACRAE, K. (1986). Importance of primary site in assessing chemotherapy response and 7-year survival data in advanced squamous cell carcinomas of the head and neck treated with initial combination chemotherapy without cisplatin. *J. Clin. Oncol.*, **4**, 1340–1347.

HO, J.H.C. (1978). An epidemiological and clinical study of nasopharyngeal carcinoma. *Int. J. Radiat. Oncol. Biol. Phys.*, **4**, 183–189.

HUANG, S.C. (1980). Nasopharyngeal cancer: a review of 1605 patients treated radically with cobalt 60. *Int. J. Radiat. Oncol. Biol. Phys.*, **6**, 401–407.

HUANG, S.C. & CHU, G.L. (1981). Nasopharyngeal cancer: study II. *Int. J. Radiat. Oncol. Biol. Phys.*, **7**, 713–716.

MERINO, O.R., LINDBERG, R.D. & FLETCHER, G.H. (1977). An analysis of distant metastases from squamous cell carcinoma of the upper respiratory and digestive tracts. *Cancer*, **40**, 145–151.

MERLANO, M., GRIMALDI, A., BRUNETTI, I., MODENISI, M., SCALA, M., MARGARINO, G., SCASSO, F., SANTELLI, A., CASTIGLIA, G., PALLESTRINI, E. & ROSSO, R. (1987). Simultaneous cisplatinum and 5-fluorouracil as second line treatment of head and neck cancer. *Cancer Treat. Rep.*, **71**, 485–488.

PALMERI, S., GEBBIA, V., RUSSO, A., GEBBIA, N., OLIVERI, D. & RAUSA, L. (1989). Cisdiamminodichloroplatinum plus 5-day continuous infusion of 5-fluorouracil in the treatment of locally recurrent and metastatic head and neck cancer patients. *J. Cancer Res. Clin. Oncol.*, **115**, 579–582.

PEREZ, C.A. & BRADY, L.W. (1987). *Principles and Practice of Radiation Oncology*. J.B. Lippincott: Philadelphia, p. 479–498.

TANNOCK, I., PAYNE, D., CUMMINGS, B., HEWITT, K. & PANZARELLA, T. (1987). Sequential chemotherapy and radiation for nasopharyngeal cancer: absence of long term benefit despite a high rate of tumor response to chemotherapy. *J. Clin. Oncol.*, **5**, 629–634.

VIKRAM, B., MISHRA, U.B., STRONG, E.W. & MANOLATOS, S. (1985). Patterns of failure in carcinoma of the nasopharynx: I. Failure at the primary site. *Int. J. Radiot. Oncol. Biol. Phys.*, **11**, 1455–1459.

VIKRAM, B., MISHRA, U.B., STRONG, E.W. & MANOLATOS, S. (1986). Patterns of failure in carcinoma of the nasopharynx: failure at distant sites. *Head Neck Surg.*, **8**, 276–279.

YATES, J.W., CHALMER, B. & MCKEGNEY, F.P. (1980). Evaluation of patients with advanced cancer using the Karnofsky performance status. *Cancer*, **45**, 2220–2224.